# Supplementary material for: Ex Vivo Evaluation of Mucosal Responses to Vaccination with ALVAC and AIDSVAX of Non-Human Primates
Source: Vaccines (Basel). 2022 Jan 25;10(2):187. doi: 10.3390/vaccines10020187 (PMC8879115; doi:10.3390/vaccines10020187)
Supplement: Supplementary file 1 [file Figure S8.pdf › Figure S8.pdf]

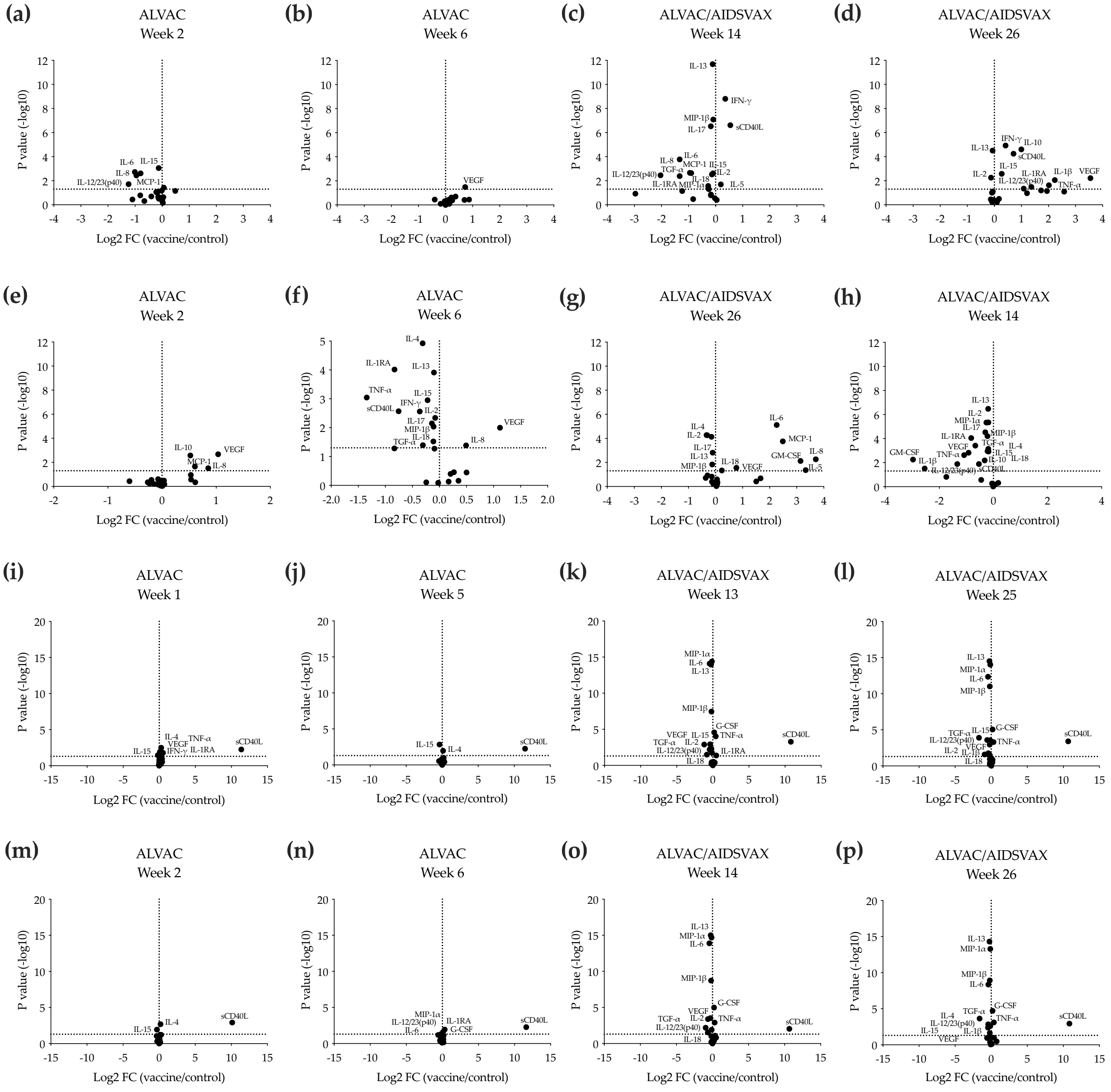

Figure S8. Modulation of mucosal and systemic cytokine profiles by ALVAC and ALVAC/AIDSVAX. Volcano plots of cytokines measured 1 or 2 weeks after each vaccination in (**a, b, c, d**) cervical and (**e, f, g, h**) rectal culture supernatants or (**i, j, k, l, m, n, o, p**) in serum in comparison to cytokine levels measured at baseline week -4 (control). FC: fold change. Horizontal dotted line indicates a P value of 0.05. Analytes above this line are significantly modulated. Vertical dotted line is set at a $Log_2FC = 0$. Data shown are mean from triplicates for each macaque (n=6).
