# Supplementary material for: Ex Vivo Evaluation of Mucosal Responses to Vaccination with ALVAC and AIDSVAX of Non-Human Primates
Source: Vaccines (Basel). 2022 Jan 25;10(2):187. doi: 10.3390/vaccines10020187 (PMC8879115; doi:10.3390/vaccines10020187)
Supplement: Supplementary file 1 [file vaccines-10-00187-s001.zip › Figure S11.pdf]

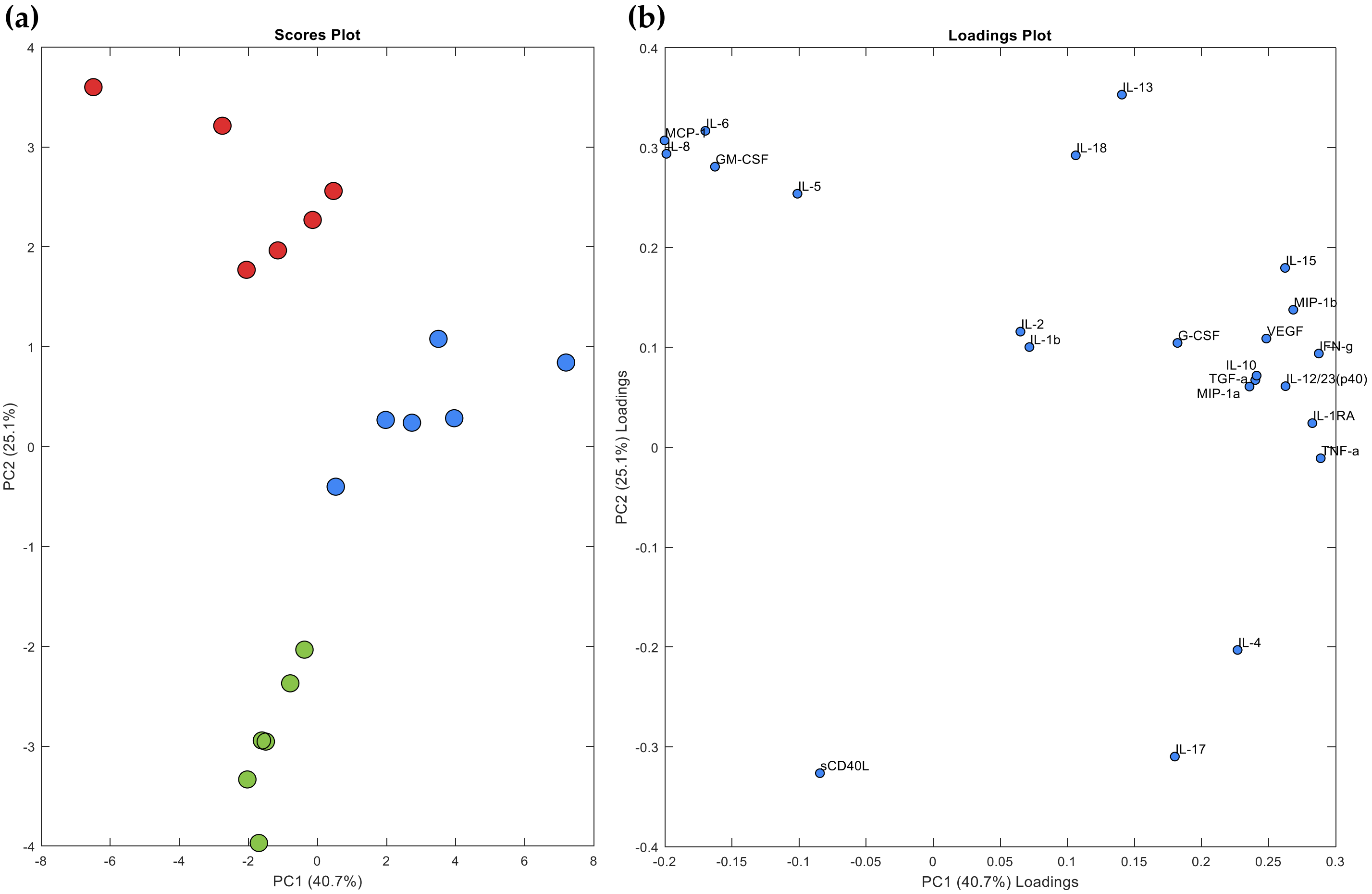

Figure S11. Analysis of cytokine signatures after the second boost with ALVAC/AIDSVAX. (**a**) PCA scores of cytokines measured in cervical explants (●), rectal tissue (●) and serum (●) at week 26; and (**b**) loading plots. The first PC accounted for 40.7 % of total variance and the second 25.1%.
