# Supplementary material for: Ex Vivo Evaluation of Mucosal Responses to Vaccination with ALVAC and AIDSVAX of Non-Human Primates
Source: Vaccines (Basel). 2022 Jan 25;10(2):187. doi: 10.3390/vaccines10020187 (PMC8879115; doi:10.3390/vaccines10020187)
Supplement: Supplementary file 1 [file vaccines-10-00187-s001.zip › Figure S12.pdf]

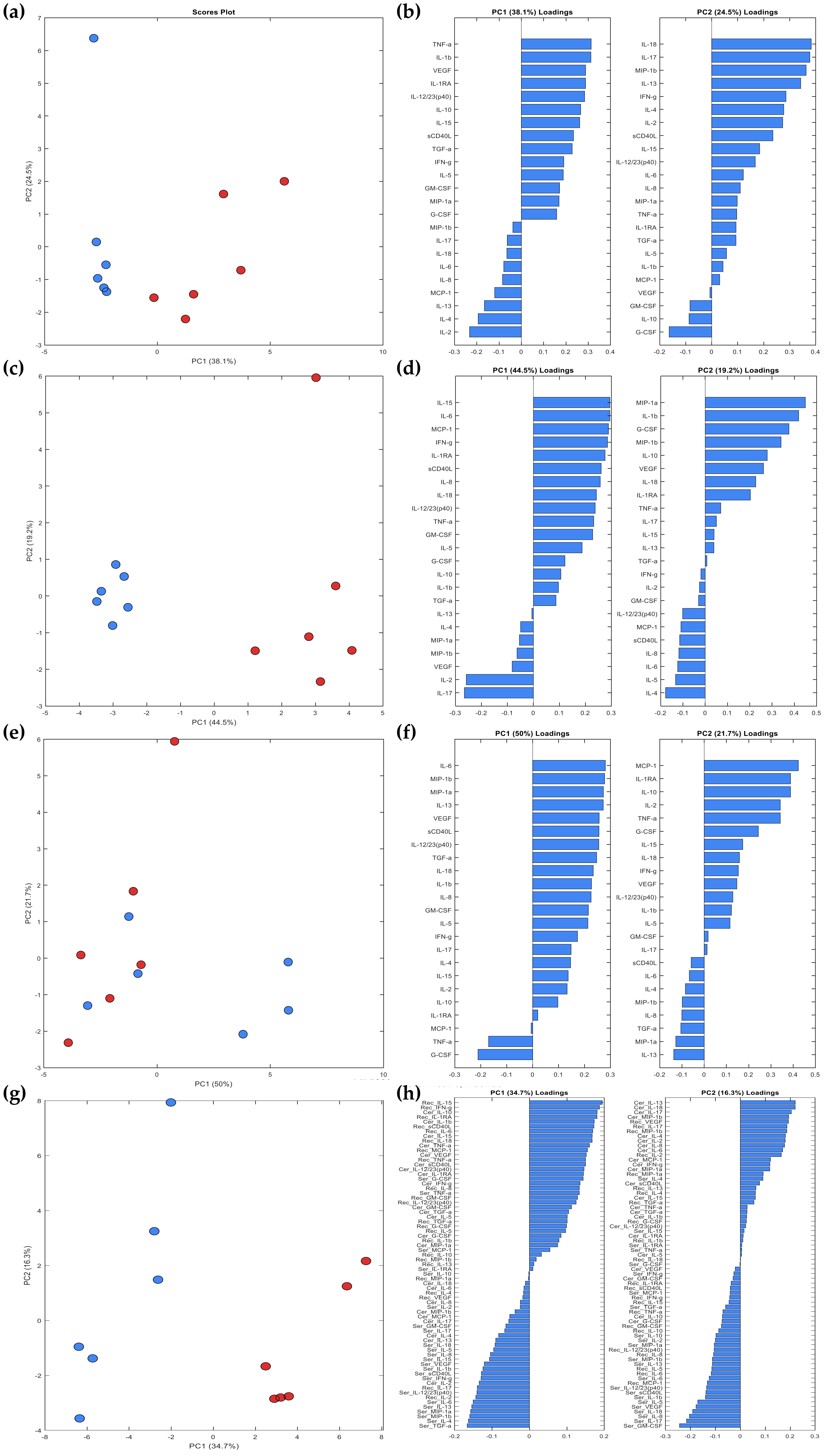

Figure S12. PCA of cytokine signatures after ALVAC and ALVAC/AIDSVAX vaccination. PCA scores of cytokines and corresponding loading plots measured in (**a**,**b**) cervical explants, (**c**,**d**) rectal explants, (**e**,**f**) serum and (**g**,**h**) in all compartments at week 6 (●) and week 26 (●).
