# Supplementary material for: Ex Vivo Evaluation of Mucosal Responses to Vaccination with ALVAC and AIDSVAX of Non-Human Primates
Source: Vaccines (Basel). 2022 Jan 25;10(2):187. doi: 10.3390/vaccines10020187 (PMC8879115; doi:10.3390/vaccines10020187)
Supplement: Supplementary file 1 [file vaccines-10-00187-s001.zip › Figure S13.pdf]

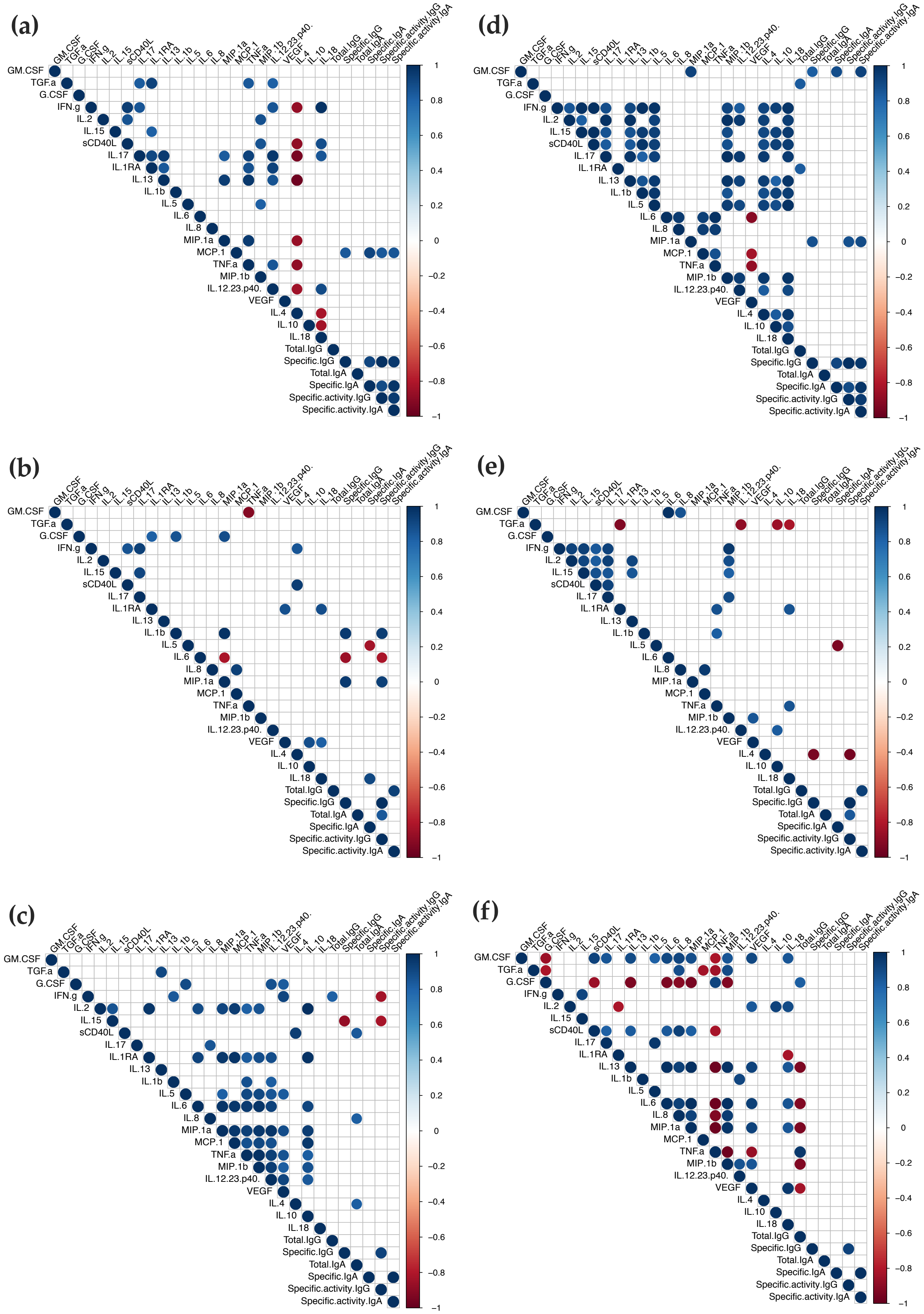

Figure S13. Correlogram of significant vaccine responses elicited in mucosal and systemic compartments. Significant ($p < 0.05$) correlations between levels of secreted cytokines and concentrations of total, gp120-specific and specific activity of IgG and IgA, in cervical tissue (**a**,**d**), rectal explants (**b**,**e**) and serum (**c**,**f**) at week 26 (**a**,**b**,**c**) or week 6 (**d**,**e**,**f**) were analyzed by a correlation matrix. Positive Pearson correlations coefficients are displayed in blue and negative correlations in red color. Color intensity indicated in the side bar is proportional to correlation coefficient. Size of the circle is proportional to the statistical significance of the correlation. Statistical significance from $p \leq 0.05$.
