# Supplementary material for: Ex Vivo Evaluation of Mucosal Responses to Vaccination with ALVAC and AIDSVAX of Non-Human Primates
Source: Vaccines (Basel). 2022 Jan 25;10(2):187. doi: 10.3390/vaccines10020187 (PMC8879115; doi:10.3390/vaccines10020187)
Supplement: Supplementary file 1 [file vaccines-10-00187-s001.zip › Figure S14.pdf]

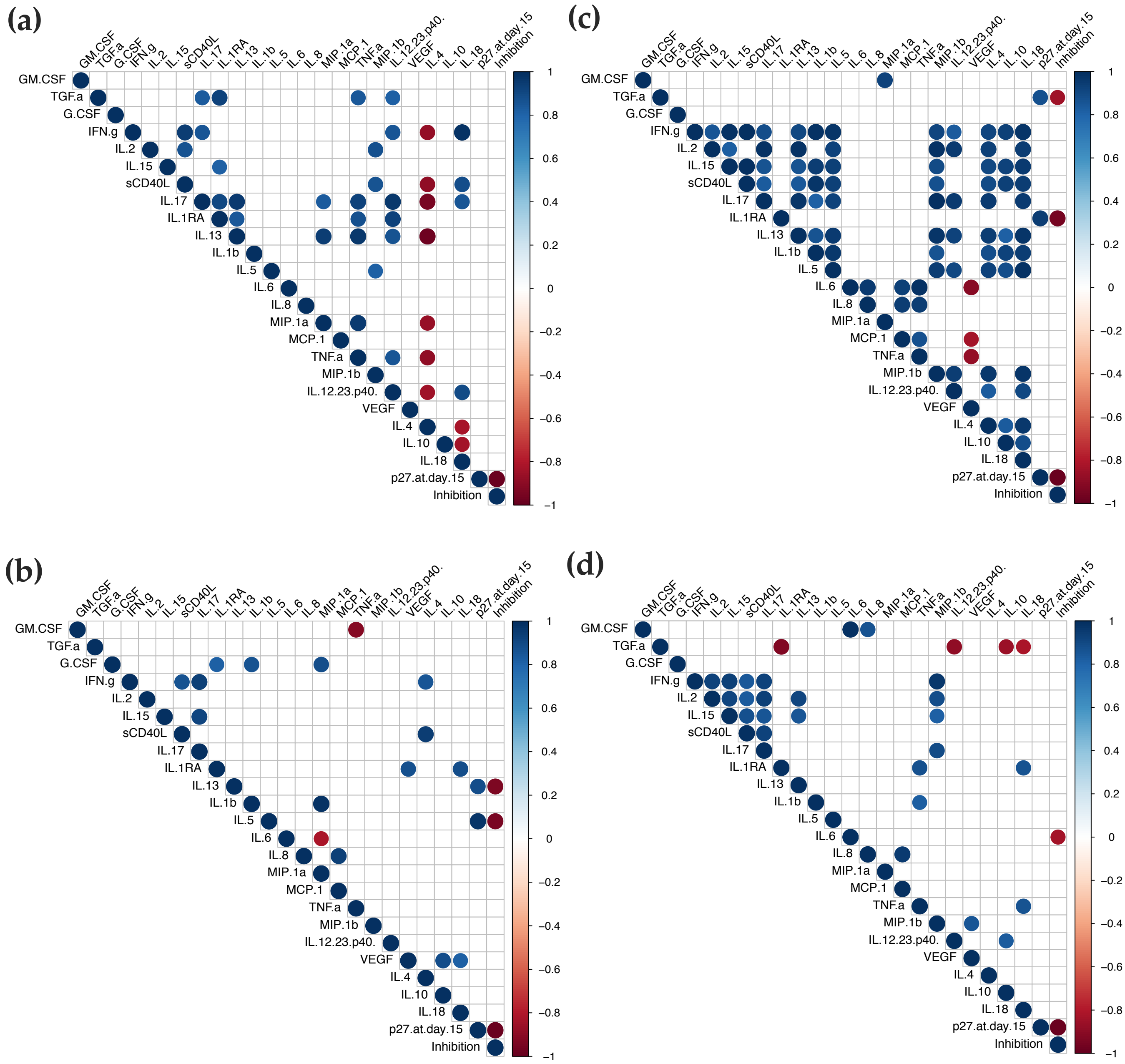

Figure S14. Correlogram of significant vaccine responses elicited in mucosal explants. Significant ($p < 0.05$) **c**orrelations between levels of secreted cytokines and susceptibility to ex vivo challenge with SHIV$_{BaL}$ ([p27] (pg/ml) at day 15 and % of inhibition) in cervical (**a**,**c**) and rectal explants (**b**,**d**) at week 26 (**a**,**b**) or week 6 (**c**,**d**) were analyzed by a correlation matrix. Positive Pearson correlations coefficients are displayed in blue and negative correlations in red color. Color intensity indicated in the side bar is proportional to correlation coefficient. Size of the circle is proportional to the statistical significance of the correlation. Statistical significance from $p \leq 0.05$.
