# Supplementary material for: Ex Vivo Evaluation of Mucosal Responses to Vaccination with ALVAC and AIDSVAX of Non-Human Primates
Source: Vaccines (Basel). 2022 Jan 25;10(2):187. doi: 10.3390/vaccines10020187 (PMC8879115; doi:10.3390/vaccines10020187)
Supplement: Supplementary file 1 [file vaccines-10-00187-s001.zip › Figure S2.pdf]

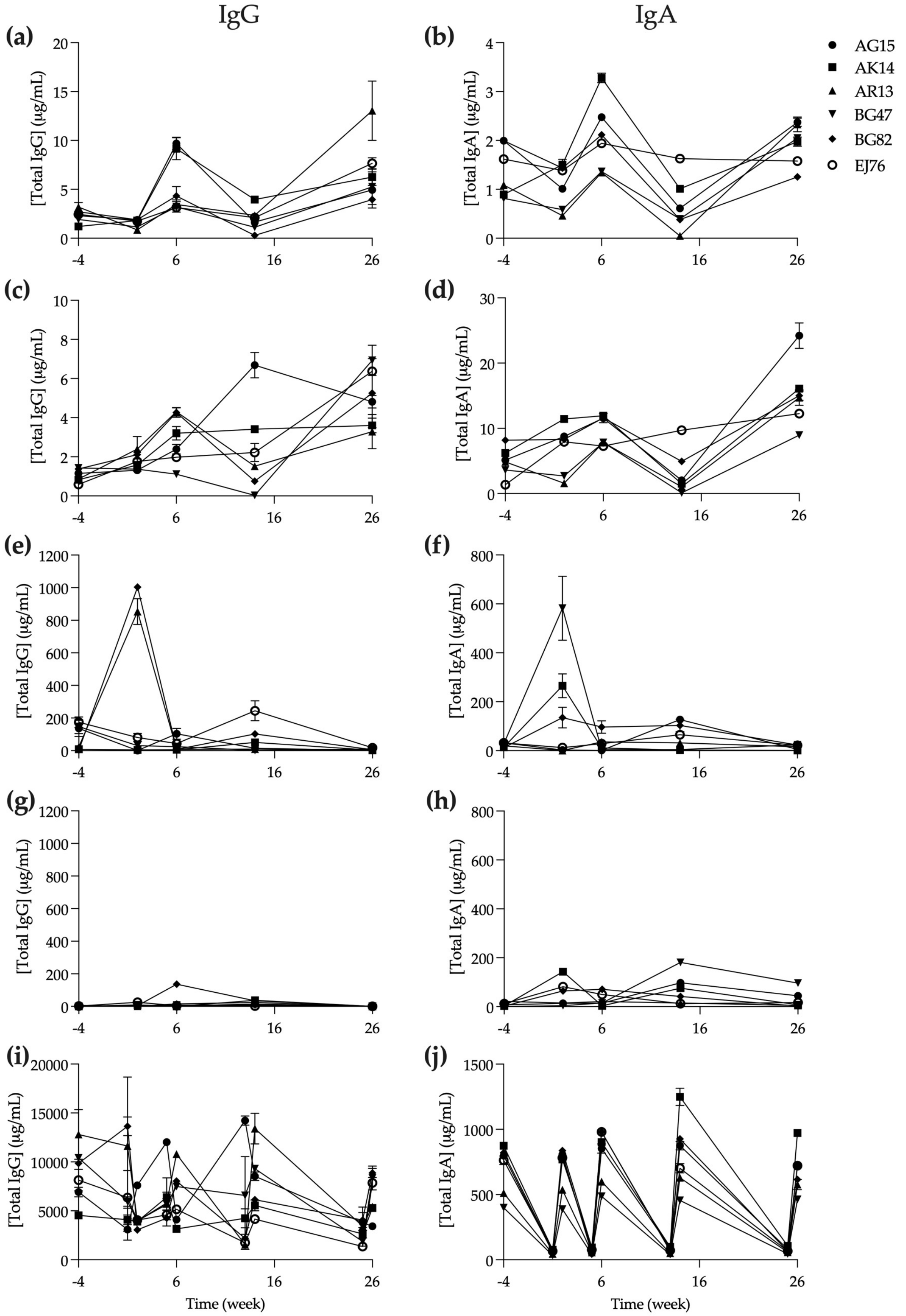

Figure S2. Total IgG and IgA levels elicited during vaccination with ALVAC/AIDSVAX for each animal. Ab concentrations were measured for each animal from (**a-d**) explant culture supernatants [(**a,b**) cervical; (**c,d**) rectal]; (**e,f**) vaginal and (**g,h**) rectal secretions; and (**i,j**) serum. Data shown are mean ± SEM from triplicates.
