# Supplementary material for: Ex Vivo Evaluation of Mucosal Responses to Vaccination with ALVAC and AIDSVAX of Non-Human Primates
Source: Vaccines (Basel). 2022 Jan 25;10(2):187. doi: 10.3390/vaccines10020187 (PMC8879115; doi:10.3390/vaccines10020187)
Supplement: Supplementary file 1 [file vaccines-10-00187-s001.zip › Figure S3.pdf]

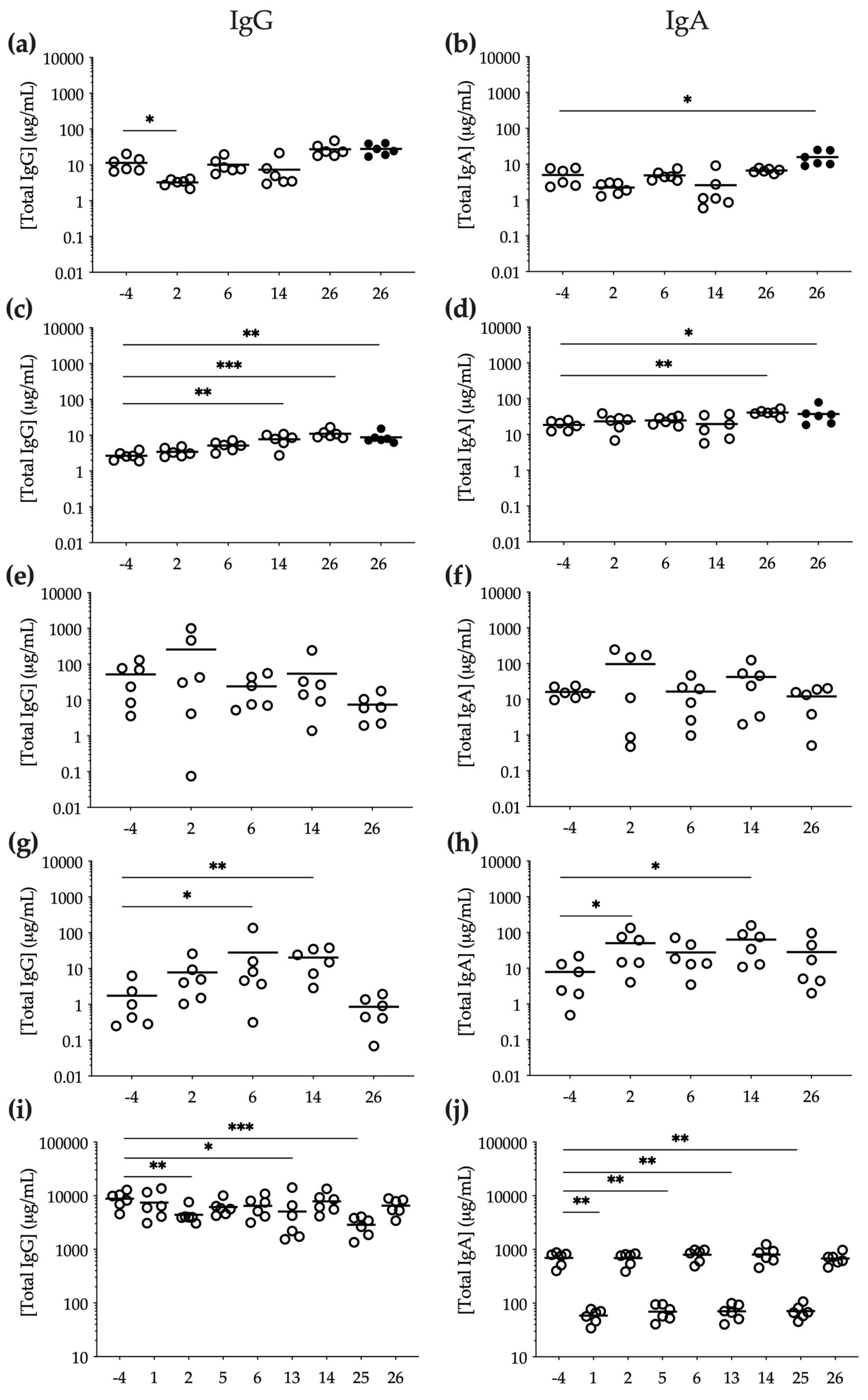

Figure S3. Total IgG and IgA levels elicited during vaccination with ALVAC/AIDSVAX. (**a-d**) Explant culture supernatants were harvested during the 15 days of culture of tissues obtained 2 weeks after each vaccination [(**a,b**) cervical (open symbols) and vaginal (closed symbols); (**c,d**) rectal (open symbols) and sigmoidal (closed symbols)]. Data shown are the sum of the Ab concentrations measured in triplicate at harvest days 3, 7, 11 and 15 during the culture period for tissue explants from each macaque. (**e,f**) Vaginal and (**g,h**) rectal secretions were extracted from Weck-Cel sponges collected from each animal 2 weeks after each vaccination and Ab concentrations were measured in triplicate. (**i,j**) Serum was obtained for each animal from blood collected 1 and 2 weeks after each

vaccination and Ab concentrations were quantified in triplicate. Means are shown with lines. Statistical significance: * $p \leq 0.05$, ** $p \leq 0.01$, *** $p \leq 0.001$, **** $p \leq 0.0001$.
