# Supplementary material for: Ex Vivo Evaluation of Mucosal Responses to Vaccination with ALVAC and AIDSVAX of Non-Human Primates
Source: Vaccines (Basel). 2022 Jan 25;10(2):187. doi: 10.3390/vaccines10020187 (PMC8879115; doi:10.3390/vaccines10020187)
Supplement: Supplementary file 1 [file vaccines-10-00187-s001.zip › Figure S4.pdf]

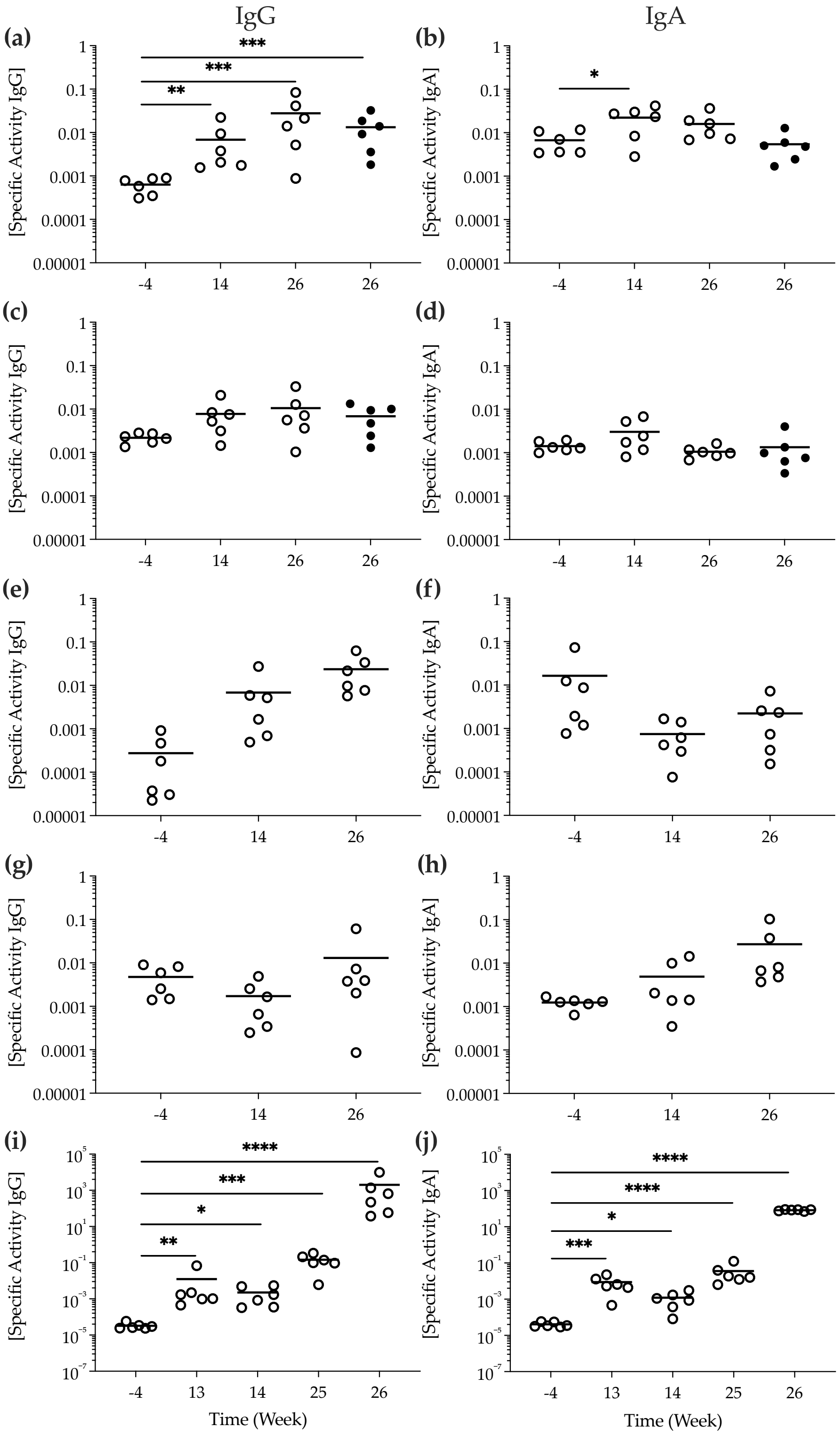

Figure S4. Specific activity of IgG and IgA during vaccination with ALVAC/AIDSVAX. The ratio of Ag-specific Ig/total Ig was calculated at baseline and after each vaccination for IgG and IGA in (**a**,**b**) cervical (open symbols) and vaginal (closed symbols) explants; (**c**,**d**) rectal (open symbols) and sigmoidal (closed symbols) explants; (**e**,**f**) vaginal and (**g**,**h**) rectal secretions; and (**i**,**j**) serum for each animal. Means are shown with lines. Statistical significance: * $p \leq 0.05$, ** $p \leq 0.01$, *** $p \leq 0.001$, **** $p \leq 0.0001$.
