# Supplementary material for: Ex Vivo Evaluation of Mucosal Responses to Vaccination with ALVAC and AIDSVAX of Non-Human Primates
Source: Vaccines (Basel). 2022 Jan 25;10(2):187. doi: 10.3390/vaccines10020187 (PMC8879115; doi:10.3390/vaccines10020187)
Supplement: Supplementary file 1 [file vaccines-10-00187-s001.zip › Figure S7.pdf]

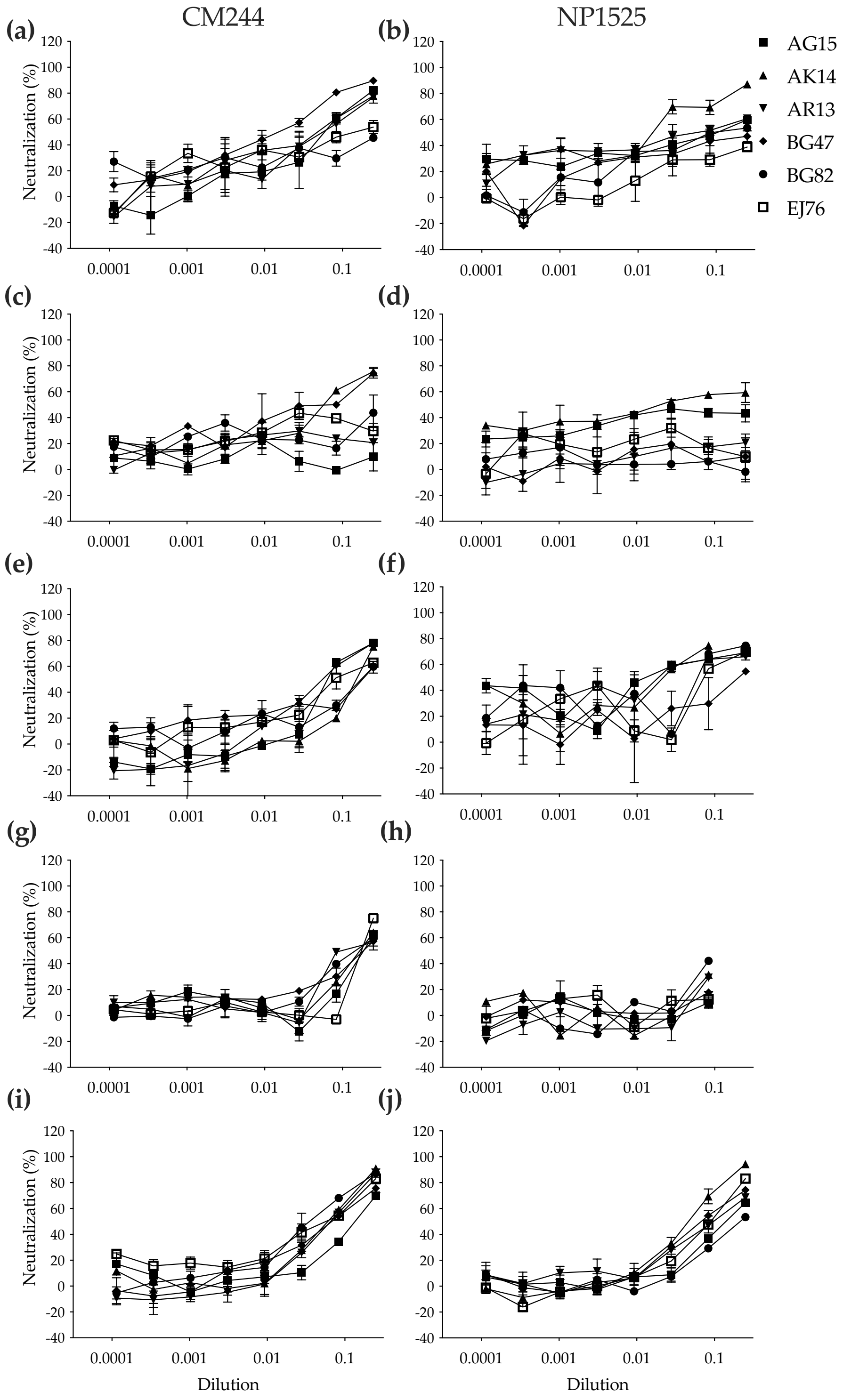

Figure S7. Neutralization potency of Abs elicited after vaccination with ALVAC/AIDSVAX. (**a, b**) Cervical and (**c, d**) rectal explant culture supernatants; (**e,f**) vaginal and (**g,h**) rectal secretion; or (**i,j**) serum from specimens obtained at week 26 were incubated in serial dilutions with HIV-1$_{CM244}$ or HIV-1$_{NP1525}$ prior to addition on TZM-bl cells. Luciferase expression (r.l.u. values) in cell lysates was determined after 48 h and the extent of neutralization was calculated. The percentage of neutralization was normalized relative to the r.l.u values obtained for cells grown in the absence of virus (100% neutralization) and for cells infected only with virus (0% neutralization). Dose-response neutralization curves are shown for each macaque as mean (SEM) from duplicates.
