# Supplementary material for: Ex Vivo Evaluation of Mucosal Responses to Vaccination with ALVAC and AIDSVAX of Non-Human Primates
Source: Vaccines (Basel). 2022 Jan 25;10(2):187. doi: 10.3390/vaccines10020187 (PMC8879115; doi:10.3390/vaccines10020187)
Supplement: Supplementary file 1 [file vaccines-10-00187-s001.zip › Figure S9.pdf]

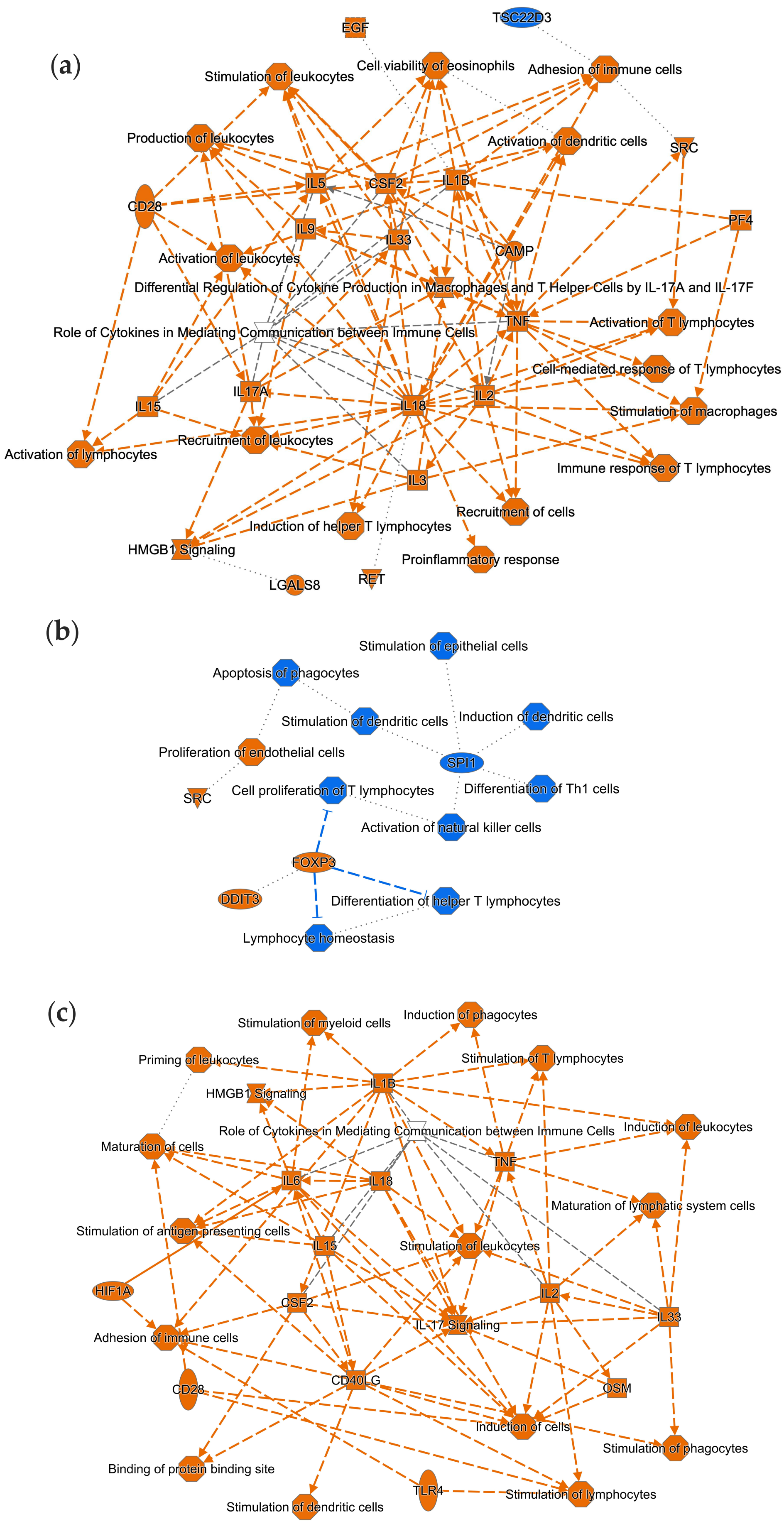

Figure S9. Top biological networks associated with two ALVAC vaccinations. Interaction maps of functional pathways associated with cytokine/chemokine nodes modulated at week 6 in (a) cervical explants, (**b**) rectal tissue or (**c**) serum. Colors: blue: downregulated; orange: upregulated. Symbols: oval: transmembrane receptor; square: cytokine/chemokine; diamond: enzyme; inverted triangle: kinase; hourglass: canonical pathway; octagon: function. Lines: solid: direct interaction; dashed: indirect interaction; doted: inferred correlation from Ingenuity Pathway Analysis machine-based learning.
