# Supplementary material for: Ex Vivo Evaluation of Mucosal Responses to Vaccination with ALVAC and AIDSVAX of Non-Human Primates
Source: Vaccines (Basel). 2022 Jan 25;10(2):187. doi: 10.3390/vaccines10020187 (PMC8879115; doi:10.3390/vaccines10020187)
Supplement: Supplementary file 1 [file vaccines-10-00187-s001.zip › Supplementary Material and Methods.pdf]

S2. Supplementary Materials and Methods

S2.1. Reagents

Monoclonal Ab (mAb) 2G12 was acquired from Polymun Scientific (Austria). Expression vector for IgG1 b6 (b6) was obtained from IAVI Neutralizing Ab Consortium and produced in house. Expression vectors for VRC01 and PGT151 were generated in house. In house mAbs were produced in HEK293T.17 cells and purified on HiTrap protein A HP column (GE LifeScience, Chicago, IL).

S2.2. Cell culture conditions

HEK293T.17 cells (ATCC, Manassas, VA) were grown in Dulbecco's Minimal Essential Medium (DMEM) (Sigma-Aldrich, Inc., St. Louis, MO) containing 10% fetal calf serum (FCS), 2 mM L-glutamine and antibiotics (100 U of penicillin/ml, 100 μg of streptomycin /ml). Cells were tested for mycoplasma contamination and confirmed mycoplasma-free.

S2.3. Tissue Explants

Surgically resected specimen of human colorectal tissue was collected at St. Mary's Hospital, Imperial College Healthcare NHS Trust, London, UK. The specimen was collected after receiving signed informed consent from the patient through the Imperial College Healthcare Tissue Bank approved by Research Ethics Committee Wales (IRAS 17/WA/0161). The patient was HIV-negative. The tissue was sectioned into 2-3 mm^3 explants comprising both epithelial and muscularis mucosae. Explants were maintained with DMEM containing 10% fetal calf serum, 2 mM L-glutamine and antibiotics (100 U of penicillin/ml, 100 μg of streptomycin/ml, 80 μg of gentamicin/ml), at 37 °C in an atmosphere containing 5% CO_2. Tissue explants were spiked with 1 μg/ml of mAb for 2 h in a non-polarized system and then washed 4 times with PBS to remove unbound mAb. Explants were then transferred onto gelfoam rafts (Welbeck Pharmaceuticals, UK) and cultured for 15 days with approximately 50% of the supernatants harvested every 2 to 3 days and replaced with fresh media. Supernatants were used for neutralization activity analysis in TZM-bl cells.
